# Supplementary material for: COZOID: contact zone identifier for visual analysis of protein-protein interactions
Source: BMC Bioinformatics. 2018 Apr 6;19:125. doi: 10.1186/s12859-018-2113-6 (PMC5889581; doi:10.1186/s12859-018-2113-6)
Supplement: Supplementary file 2 — Software build. Executable binary file of the software tool. (ZIP 118,784 kb) [file 12859_2018_2113_MOESM2_ESM.zip › cozoid/java/docs/beansbinding-1.2.1-doc.zip!/org/jdesktop/beansbinding/Validator.html]

Validator (Beans Binding)


---


|  |  |  |
| --- | --- | --- |
| **Overview** \| **Package** \| **Class** \| **Use** \| **Tree** \| **Deprecated** \| **Index** \| **Help** | |  |
| **PREV CLASS**   **NEXT CLASS** | **FRAMES**    **NO FRAMES**     **All Classes** |  |
| SUMMARY: NESTED \| FIELD \| CONSTR \| METHOD | DETAIL: FIELD \| CONSTR \| METHOD |  |


---


## org.jdesktop.beansbinding Class Validator<T>

```
java.lang.Object
  org.jdesktop.beansbinding.Validator<T>
```

**Type Parameters:**: `T` - the type of value that this validator can validate

---

``` public abstract class Validator<T> extends java.lang.Object ```

`Validator` is responsible for validating the value from the target of
a `Binding`.

---

| **Nested Class Summary** | |
| --- | --- |
| `class` | `Validator.Result`             An instance of `Result` is returned from a `Validator's` `validate` method to indicate an invalid value. |


**Constructor Summary**

`Validator()`


| **Method Summary** | |
| --- | --- |
| `abstract  Validator.Result` | `validate(T value)`             Validates a value; returns `null` for a valid value, and a `Result` object describing the problem for an invalid value. |

**Methods inherited from class java.lang.Object**

`clone, equals, finalize, getClass, hashCode, notify, notifyAll, toString, wait, wait, wait`

**Constructor Detail**

### Validator

```
public Validator()
```


**Method Detail**

### validate

```
public abstract Validator.Result validate(T value)
```

:   Validates a value; returns `null` for a valid value, and a
    `Result` object describing the problem for an invalid value.

    :   **Parameters:**: `value` - the value to validate, may be `null` **Returns:**: `null` for a valid value or a `Result` describing the problem for an invalid value


---


|  |  |  |
| --- | --- | --- |
| **Overview** \| **Package** \| **Class** \| **Use** \| **Tree** \| **Deprecated** \| **Index** \| **Help** | |  |
| **PREV CLASS**   **NEXT CLASS** | **FRAMES**    **NO FRAMES**     **All Classes** |  |
| SUMMARY: NESTED \| FIELD \| CONSTR \| METHOD | DETAIL: FIELD \| CONSTR \| METHOD |  |


---
